# Herbivore-induced stomatal closure in tomato is mediated by a Ca^2^⁺-dependent GLR-CPK27-SLAC1 pathway

**DOI:** 10.1186/s43897-026-00237-8

**Published:** 2026-07-06

**Authors:** Huijia Kang, Xinyi Wu, Chufan Xue, Xinyan Li, Yuhui Jia, Yanhong Zhou, Jingquan Yu, Chaoyi Hu

**Affiliations:** 1 Department of Horticulture, Zhejiang University, Zijingang Campus866 Yuhangtang Road, Hangzhou, 310058 People’s Republic of China; 2 Hainan Institute, Zhejiang University, Sanya, 572025 People’s Republic of China; 3 Zhejiang Key Laboratory of Horticultural Crop Quality Improvement, 866 Yuhangtang Road, Hangzhou, 310058 People’s Republic of China

Insect herbivores impose significant constraints on crop yield and quality, accounting for up to 80% of global production losses in pre-harvest yields of major crops (Koch et al. 2016). It is well documented that herbivory impacts plant growth not only through loss of leaf area but also by reducing photosynthesis in remaining tissues (Zangerl et al. 2002). Stomata, microscopic pores formed by paired guard cells in terrestrial plants, serve as the primary gateways for atmospheric CO₂ uptake and transpirational water loss. As central regulators of photosynthesis and transpiration, they enable plants to rapidly respond to environmental cues and play pivotal roles in both biotic and abiotic stress responses. Increasing evidence suggests that herbivore attack, such as from spider mites (*T. urticae* Koch), can trigger stomatal closure (Schmidt et al. 2009), which may account for the reduction in photosynthesis due to herbivory. However, the molecular mechanisms underlying herbivore-triggered stomatal regulation remain unclear. In this study, we employ the *Helicoverpa armigera* (cotton bollworm)-*Solanum lycopersicum* (tomato) interaction as a model system to investigate how plants integrate early signaling events, such as electrical signals, Ca^2^⁺ fluxes, reactive oxygen species (ROS) bursts, and defense hormone signaling, into a cascading network that modulates stomatal movement and orchestrates broader defense responses.

We established a feeding system using cotton bollworms to assess their effect on stomatal dynamics in tomato. Consistent with prior studies (Schmidt et al. 2009), larval feeding caused a significant reduction in stomatal conductance and aperture in tomato leaves within 15 min, with the inhibitory effect increasing after 30 min of feeding (Fig. 1A-B). Simulated herbivory, achieved by mechanically wounding both sides of the leaf followed by the application of 10 μL of one-fifth-diluted cotton bollworm oral secretions (W + OS), also led to a reduction in stomatal conductance and aperture within 15 min (Fig. 1C-D), confirming the role of feeding-specific signals in triggering stomatal closure. The abscisic acid (ABA) and wound-induced jasmonic acid (JA) signaling pathways are reported to regulate stomatal movement in Arabidopsis (Fötster et al. 2019; Hsu et al. 2021; Mousavi et al. 2013; Munemasa et al. 2007), and they also play a critical role in mediating responses to bacterial infection in tomato (Du et al. 2014). We then examined the relationship betwen ABA and JA on herbivore-induced stomatal closure. Simulated herbivory significantly increased JA accumulation within 15 min after the W + OS treatment, while ABA levels remained unchanged (Fig. 1E-1F). Notably, the stomatal aperture in the JA-insensitive mutant (*jai1*) and the JA transcription factor mutant (*myc2*) showed no significant difference from the wild type (WT) (Fig. 1G-1H and Supplemental Fig. 1A-1B), particularly *myc2,* which exhibited no difference even at earlier time points (Supplemental Fig. 1C-1D). These results indicate that although JA biosynthesis is induced upon herbivore attack, JA signaling does not serve as the main regulator of stomatal closure. In addition, the ABA-deficient mutant (*not*) exhibited significantly higher stomatal conductance than WT under both mock and W + OS conditions, confirming the critical role of ABA in regulating stomatal conductance (Fig. 1I and Supplementary Fig. 1E). However, similar to WT, *not* mutants still exhibited a significant reduction in stomatal conductance following W + OS treatment, indicating that ABA also is not the primary mediator of herbivory-induced stomatal closure. This prompted us to investigate whether alternative early signaling events during herbivory might mediate this rapid stomatal closure response.

**Fig. 1:**
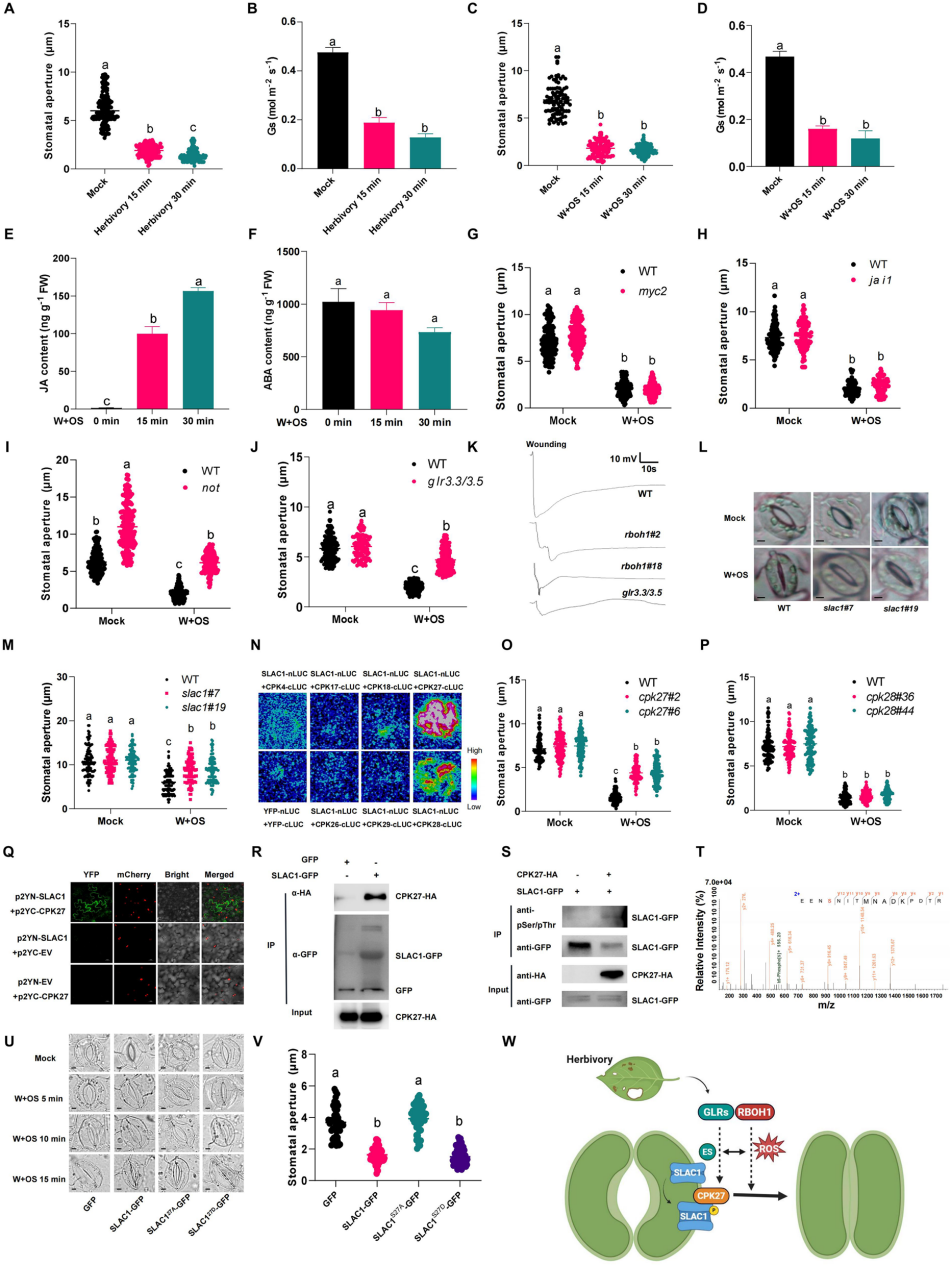
Electrical, Ca.^2^⁺ and ROS signals jointly regulate the stomatal closure process in tomato herbivory response. Stomatal aperture of tomato leaves after 15 and 30 min (min) of herbivory by *H*. *armigera*. Data represent the mean ± standard deviation (SD). (*n* > 100). **B** Stomatal conductance of tomato leaves after 15 and 30 min of herbivory by *H*. *armigera*. Data represent the mean ± SD. (*n* = 6). **C** Stomatal aperture of tomato leaves after 15 and 30 min of simulated herbivory (W + OS). Data represent the mean ± SD. (n > 100). **D** Stomatal conductance of tomato leaves after 15 and 30 min of W + OS. Data represent the mean ± SD. (*n* = 6). **E**, **F** Accumulation of JA (**E**) and ABA (**F**) after simulated herbivory treatment. Data represent the mean ± SD (*n* = 4). **G**, **H** Stomatal aperture of WT, *myc2* (**G**) and *jai1* (H) tomato leaves after W + OS. Data represent the mean ± SD. (*n* > 100). **I** Stomatal aperture of WT and *not* tomato leaves after W + OS. Data represent the mean ± SD. (*n* > 100). **J** Stomatal aperture of WT and *glr3.3/3.5* tomato leaves after W + OS. Data represent the mean ± SD (*n* > 100). **K** Effect of wounding on the surface potential in *glr3.3/3.5*, *rboh1,* and WT leaves. **L** Representative images of stomata in WT and *slac1#7/#19* leaves after W + OS. Scale bar = 5 μm. (M) Stomatal aperture of WT and *slac1* tomato leaves after W + OS. Data represent the mean ± SD. (*n* > 100). **N** Split-luciferase assay screening for CPKs interacting with SLAC1. **O** Stomatal aperture of WT and *cpk27* tomato leaves after W + OS. Data represent the mean ± SD. (*n* > 100). **P** Stomatal aperture of WT and *cpk28* tomato leaves after W + OS. Data represent the mean ± SD. (*n* > 100). Bimolecular fluorescence complementation (BiFC) analyses (**Q**) and co-immunoprecipitation (Co-IP) assays (**R**) demonstrating the interaction between CPK27 and SLAC1. **S** Phosphorylation of SLAC1 by CPK27 in vivo*.* Co-expression of CPK27-HA and SLAC1-GFP in *Nicotiana benthamiana*, as detected by anti-GFP immunoprecipitation. A GFP vector was used as a negative control. **T** LC–MS/MS analysis showing that S27 in SLAC1 is directly phosphorylated by CPK27. Serine (**S**) residues in the SLAC1 fragment are highlighted in red. **U** Representative images of stomata in GFP, SLAC1-GFP, SLAC127A-GFP, and SLAC127D-GFP after 0, 5, 10, and 15 min of W + OS treatment. Scale bar = 5 μm. **V** Stomatal aperture of GFP, SLAC1-GFP, SLAC127A-GFP, and SLAC127D-GFP after 10-min W + OS treatment. Data represent the mean ± SD. (*n* > 50). **W** Schematic model illustrating plant stomatal responses to herbivory: Upon herbivory, stomatal closure is triggered through the interaction of electrical signals mediated by GLR3.3/3.5, Ca2⁺, and RBOH1-dependent ROS signaling. In addition, calcium-dependent protein kinase CPK27 phosphorylates SLAC1, thereby promoting rapid stomatal closure. This figure was generated using https://BioRender.com. Different letters indicate statistically significant differences between treatments (*P* < 0.05, one-way or two-way ANOVA followed by Tukey's test)

Given that reactive oxygen species (ROS) and calcium (Ca^2^⁺) fluxes are among the earliest stress signals following herbivory, we investigated their potential roles in regulating stomata (Zandalinas and Mittler 2021). We observed that both cotton bollworm feeding and W + OS treatment induced ROS accumulation in the tissues surrounding the wound (Supplemental Fig. 2A-B) and guard cells (Supplemental Fig. 2C-D). Importantly, the *rboh1* mutant, which is defective in the ROS-producing enzyme respiratory burst oxidase homolog 1, exhibited significantly reduced stomatal closure following W + OS treatment compared to wild-type plants (Supplemental Fig. 2E-2G). This confirmed that the ROS signal generated by RBOH1 is essential for stomatal closure in response to herbivore-associated cues.

Ca^2^⁺ ions function as a ubiquitous secondary messenger that rapidly responds to environmental stimuli. To determine whether Ca^2^⁺ signaling contributes to the reduction of stomatal aperture induced by herbivory, tomato leaves were pretreated with the calcium channel inhibitors ruthenium red (RR) and verapamil (Ver). This pretreatment completely abolished the W + OS-induced stomatal closure, and the stomatal aperture in the inhibitor-treated group was significantly higher than in the untreated control (Supplemental Fig. 3A-C). To further dissect the molecular basis underlying this response, we examined the glutamate receptor-like (GLR) calcium channels, which have previously been implicated in herbivore-induced Ca^2^⁺ signaling in tomato (Hu et al. 2022). The *glr3.3/3.5* double mutant exhibited attenuated wound-induced electrical signals and significantly higher stomatal conductance following W + OS treatment compared to WT (Fig. 1J-K and Supplemental Fig. 3D-E). Together, these results demonstrate that GLR3.3/3.5-mediated Ca^2^⁺ influx is required for herbivore-induced stomatal closure.

Plant responses to herbivory involve rapid and complex signal transduction events, including electrical signals, Ca^2^⁺ fluctuations, and ROS bursts (Kloth and Dicke 2022). To investigate how these early signals interact to mediate stomatal closure, we examined ROS accumulation dynamics in WT, *rboh1*, and *glr3.3/3.5* plants. Guard cell staining showed that ROS accumulation in both mutants was significantly lower than in WT, consistent with their impaired stomatal closure phenotype (Supplemental Fig. 4A). Given that GLRs have been implicated in both Ca^2^⁺ and electrical signaling (Salvador-Recatalà, 2016), we monitored changes in membrane potential following mechanical damage. WT plants showed pronounced changes in membrane potential, while both *rboh1* and *glr3.3/3.5* mutants exhibited significantly attenuated electrical responses (Fig. 1K and Supplemental Fig. 4B). Together, GLR3.3/3.5-mediated electrical signaling, Ca^2^⁺ influx, and RBOH1-dependent ROS production form a tightly coordinated early signaling module that initiates stomatal closure in response to herbivory.

Next, we sought to identify downstream effectors responsible for executing this closure response. SLOW ANION CHANNEL 1 (SLAC1) is a key regulator of stomatal movement, facilitating anion efflux to disrupt osmotic equilibrium and induce stomatal closure (Salvador-Recatalà, 2016). To assess its role in herbivore-induced stomatal regulation, we generated *SLAC1* knockout (KO) plants using CRISPR-Cas9 (Supplemental Fig. 5). The *slac1* mutant lines exhibited significantly attenuated reductions in stomatal conductance and aperture in response to simulated feeding (Fig. L-M).

Given that SLAC1 activity can be activated by Ca^2^⁺ signals (Huang et al. 2019), we further explored its upstream regulatory factors. Calcium-dependent protein kinases (CPKs) are key calcium sensors that translate Ca^2^⁺ signals into phosphorylation cascades (Delormel and Boudsocq 2019). Following a one-hour mechanical injury treatment, the expression levels of *CPK4, 17, 18, 26, 27, 28,* and *29* in tomato were significantly upregulated (Supplementary Fig. 6A). Using split luciferase screening, we identified that SLAC1 interacts with CPK27 and CPK28 in vitro (Fig. 1N). Functional analyses revealed that stomatal aperture and conductance in the *cpk27* mutant were significantly greater than in WT after W + OS treatment (Fig. 1O and Supplementary Fig. 6B-D), whereas the *cpk28* mutant showed no significant differences from WT (Fig. 1P and Supplementary Fig. 6D-E). The interaction between CPK27 and SLAC1 was further confirmed by BiFC, Co-IP, and split luciferase assays (Fig. 1Q-R and Supplemental Fig. 7A). Next, we demonstrated that CPK27 enhanced SLAC1 phosphorylation (Fig. 1S). To map the phosphorylation sites, recombinant MBP-SLAC1 and His-CPK27 proteins were purified and subjected to in vitro phosphorylation assays. Mass spectrometry identified two CPK27-targeted phosphorylation sites on SLAC1: Serine-27 (S27) and Serine-122 (S122) (Supplementary Fig. 7B), with S27 exhibiting the highest confidence score (> 0.75) (Fig. 1T). To determine the functional significance of the S27 site in vivo, we generated phospho-null (S27A) and phospho-mimic (S27D) SLAC1 mutants fused to GFP. Following W + OS treatment, plants expressing SLAC1-GFP showed a significant reduction in stomatal aperture relative to GFP-only controls, while this response was abolished in the SLAC1 S27A-GFP line (Fig. 1U-V). Notably, the expression of *CPK27* was suppressed in the *glr3.3/3.5* mutant after W + OS treatment (Supplemental Fig. 6F), indicating that GLR3.3/3.5 are essential components within the CPK27 cascade involved in regulating stomatal movement.

Based on the above findings, we propose a regulatory model for herbivore-induced stomatal closure in tomato (Fig. 1W). In this model, the SLAC1 anion channel functions as the central effector, facilitating stomatal closure in response to insect feeding. This process is governed by a coordinated signaling network that integrates GLR3.3/3.5-mediated electrical signals, Ca^2^⁺ influx, and RBOH1-dependent ROS production. CPK27 functions downstream of Ca^2^⁺ signals to phosphorylate SLAC1, thereby promoting rapid stomatal closure. Importantly, both genetic and biochemical evidence indicate that this pathway operates independently of the canonical JA and ABA signaling pathways. Collectively, these findings provide new mechanistic insights into the rapid modulation of stomatal aperture in response to herbivore attack, offering a refined perspective on the balance between wound signaling-mediated defense mechanisms and plant growth.

## Supplementary Information

Supplementary Material 1.

## Data Availability

All data are available in the manuscript.
