# Supplementary material for: Herbivore-induced stomatal closure in tomato is mediated by a Ca2⁺-dependent GLR-CPK27-SLAC1 pathway
Source: Mol Hortic. 2026 Jul 6;6:50. doi: 10.1186/s43897-026-00237-8 (PMC13335355; doi:10.1186/s43897-026-00237-8)
Supplement: Supplementary file 1 — Supplementary Material 1. [file 43897_2026_237_MOESM1_ESM.docx]

# Supplemental information

**Herbivore-induced stomatal closure in tomato is mediated by a Ca²⁺-dependent GLR-CPK27-SLAC1 pathway**

This PDF file includes:

Materials and Methods

Supplemental References

Supplemental Figures 1-7

Supplemental Table 1-2

# Materials and Methods

**Plant materials and growth conditions**

The research utilized the wild type (Ailsa Craig) of tomato (*Solanum lycopersicum*), obtained from the Tomato Genetic Resource Center at the University of California, Davis, and propagated in the laboratory. Tomato seeds were sown in a substrate composed of peat and vermiculite in a 2:1 (v:v) ratio and placed in a plant factory for growth. The growth conditions were as follows: temperature of 25/20 °C (day/night), a 12-hour photoperiod, and a photosynthetic photon flux density (PPFD) of 400 μmol m^-2^ s^-1^. Hoagland nutrient solution was used for regular watering.

The wild type (Condine Red) of tomato, as well as the *CPK27* mutants *cpk27#2* and *cpk27#6*, and the *CPK28* mutants *cpk28#36* and *cpk28#44*, with Condine Red as the background, were used. The mutants *cpk27* (Zhu et al., 2024), and *cpk28* (Hu et al., 2021c) have been previously reported. The mutants *jai1* (Hu et al., 2021a), *myc2* (Hu et al., 2021a), *glr3.3/3.5* (Hu et al., 2021b), *rboh1* (Zheng et al., 2024) and have been previously reported. The *slac1* mutants were generated by CRISPR/Cas9 technology. The target sequence for *SLAC1* was designed using the web tool CRISPR-P (http://crispr.hzau.edu.cn/cgi-bin/CRISPR2/CRISPR), and the pHEE401 vector was transformed into *A. tumefaciens* strain EHA105 and subsequently introduced into tomato, as described in a previous study (Hu et al., 2022).

**Plant treatment**

Plants at the six-leaf stage were used for treatment, with the second fully expanded leaf from the top selected for experimental treatment. Fourth-instar larvae of cotton bollworm (*Helicoverpa armigera*) were used for herbivory feeding. Healthy, uniform-sized larvae were starved for 24 hours prior to placement on tomato leaves. A bug net was used to restrict the movement of the larvae to the designated leaves. The treatment began when the larvae commenced feeding, and measurements or sampling were conducted at 15 or 30 minutes post-treatment.

For simulated herbivory treatments, mechanical damage (wounding, W) was inflicted on both sides of the tomato leaves using hemostatic forceps. Immediately after injury, 10 μL of diluted (1:5) *Helicoverpa armigera* oral secretions (OS) were applied to the injured area. The oral secretions were extracted from fourth-instar larvae and stored at -80°C. The W+OS treatment was conducted for 15 or 30 minutes before measurements or sampling. All plants were maintained in their original growth environment during the treatment period.

**Measurement of stomatal conductance and aperture**

Stomatal conductance and aperture were measured on fully expanded leaves of the second leaf position of tomato plants using a LI-6400 portable photosynthesis system (LI-COR, USA) from 9:00 AM to 11:00 AM. Parameters were recorded after the leaf state stabilized. The experimental conditions were set as follows: temperature at 25°C, saturation light intensity at 800 μmol m⁻² s⁻¹, relative humidity at 80%, and atmospheric CO₂ concentration at approximately 380 μmol mol⁻¹. Four plants were measured for each treatment group.

The second fully expanded leaves of six-leaf-stage tomato plants were subjected to W+OS treatments. After 15 or 30 minutes of treatment, the lower epidermis of the leaves was removed using forceps. Stomatal aperture was observed and photographed under an optical microscope (Leica Microsystems, Germany). ImageJ software was used to count stomatal pore size in 8 fields of view. A minimum of 100 stomata were counted per treatment group.

**Leaf and guard cells ROS staining**

Reactive oxygen species (ROS) staining in tomato leaves was performed using 3,3'-diaminobenzidine (DAB) and nitroblue tetrazolium chloride (NBT) staining methods. Freshly prepared 0.1% DAB and 0.5 mg/mL NBT solutions were prepared in 50 mM Tris-HCl and 25 mM HEPES, respectively, and stored in the dark. After 15 minutes of cotton bollworm feeding and W+OS treatment, 5-10 leaves from the reverse side of 4 plants per treatment were fully immersed in the staining solution. DAB staining was incubated at 25°C under light, while NBT staining was incubated in the dark at 25°C. The staining was stopped when dark spots appeared on the leaves. The leaves were then decolorized in 95% ethanol at 95°C until chlorophyll was removed, and images were recorded.

For guard cell ROS staining was performed as described in a previous study (Shi et al., 2015). A stock solution of 50 mM H2DCF-DA (2,7-dichlorofluorescin diacetate) in dimethyl sulfoxide (DMSO) was stored at -20°C. After 15 or 30 minutes of herbivory stress and control treatment, the lower epidermis of tomato leaves was immediately removed and placed in a loading buffer (50 mM KCl, 10 mM MES, pH 6.15). H2DCF-DA was added to a final concentration of 50 μM. The mixture was incubated in the dark at room temperature (25 ± 2°C) for 10-15 minutes. The leaf epidermis was rinsed three times with the loading buffer, and ROS accumulation in guard cells was observed using a laser confocal microscope (LCM-500, Zeiss, Germany).

**Determination of hormone content**

The determination of jasmonic acid (JA) and abscisic acid (ABA) content was performed using three biological replicates, following the method described by Hu et al. (2022) (Hu et al*.*, 2022). Tomato plants were sampled 15 minutes after W+OS treatment. The samples were homogenized in liquid nitrogen and 0.1 g of tissue was weighed. Then, 1mL of chromatographic-grade ethyl acetate was added, and 100 ng of D6-ABA (C/D/N isotopes, Canada) and 50 ng of D5-JA (C/D/N isotopes, Canada) internal standards were added in the dark. The mixture was shaken at 4°C and 150 rpm in the dark overnight. After centrifugation at 18,000 g and 4°C for 10 minutes, the supernatant was collected. A second extraction was performed by adding 1 mL of chromatographic-grade ethyl acetate to the precipitate, followed by a 2-hour incubation. After centrifugation, the supernatants were combined and dried under nitrogen. The residue was dissolved in 0.5 mL of 70% chromatographic-grade methanol. The concentrations of ABA and JA were determined using a liquid chromatography-tandem mass spectrometry system (Varian 320-MS LC/MS, Agilent Technologies, Amstelveen, the Netherlands).

**Electrical signal measurement**

Electrical signal measurement was conducted on tomato seedlings with three leaves, the assay following the method as previously described (Hu et al., 2021a). The measurement was performed using an FD 223 with a dual-channel amplifier and a Duo 773 (World Precision Instruments). A silver electrode with a diameter of 0.5 mm was placed in a 10 μL droplet containing 0.5% (w/v) agar and 10 mM KCl at the junction of the tomato leaf and petiole, and the ground electrode was placed in the soil. A mechanical injury was created in the middle of the fully expanded leaf at the second leaf position of the tomato plant using a plastic hemostat, and the potential changes at the junction of the leaf and petiole were measured.

**Split-luciferase assay**

The split-luciferase assay was performed as described in a previous study (Hu et al., 2021a). The pCAMBIA-GW-nLUC and pCAMBIA-GW-cLUC vectors were used to construct SLAC1-nLUC and CPKs-cLUC, respectively. The primer sequences for vector construction are listed in Supplementary Table 1. SLAC1-nLUC and CPKs-cLUC vectors were co-transformed into *Nicotiana benthamiana* leaves. The controls included SLAC1-nLUC + cLUC empty vector, CPK27-cLUC + nLUC empty vector, and cLUC empty vector + nLUC empty vector. After 48 hours of infection, 1 mM luciferin (MedChemExpress, USA) was evenly applied to the back of the leaves. Ten minutes later, images for the split-luciferase assay were captured using a Photek camera (HRPCS5, Photek).

**Bimolecular fluorescence complementation (BiFC) assay**

The BiFC assay was conducted following the method of Yang et al. (Yang et al., 2007). The full-length CDS sequences of *CPK27* and *SLAC1* were cloned into the BiFC vectors pGTQL1221YC and pGTQL1211YN, respectively. The primers used for vector construction are listed in Supplementary Table 1. *A.* *tumefaciens* suspensions harboring the two constructs were mixed at a 1:1 ratio and injected into the leaves of *N. benthamiana* expressing *35Spro:FLS2:mCherry* as a marker using a syringe without a needle. The control was a single empty vector combination. After 48 h, the YFP and mCherry fluorescence signals were observed using a confocal laser scanning microscope (Zeiss, Germany). The excitation and emission parameters were: YFP (514/520-560 nm), mCherry (561/580-620 nm).

**Co-IP assay**

The Co-IP experiment was was performed as described in a previous study (Hu et al., 2022). Binary vectors pFGC1008-CPK27-HA and pCAMBIA2300-35S:SLAC1-GFP were constructed. The primer sequences used for vector construction are listed in Supplementary Table 1. Activated *A. tumefaciens* containing the vectors were mixed at a 1:1 ratio and co-infiltrated into *N. benthamiana* leaves for 48 h. Empty vectors were used as negative controls. Protein was extracted using 1 mL of Co-IP buffer (50 mM Tris-HCl, pH 7.5, 150 mM NaCl, 5 mM EDTA, 0.5% Triton, 1× protease inhibitor, 2.5 μL 0.4 M DTT, 2 μL 1 M NaF, 2 μL 1 M Na_3_VO_3_). A 50 μL sample of the extracted protein solution was taken as input for control. 15 μL of α-GFP magnetic beads (Chromotek, AB_2631358) were added to the protein solution. After shaking and incubating at 4 °C for 2 h, the mixture was washed four times with Co-IP washing buffer (50 mM Tris-HCl, pH 7.5, 150 mM NaCl, 5 mM EDTA, 0.1% Triton) and once with 50 mM Tris-HCl (pH 7.5). Finally, 40 μL of 2× SDS-PAGE loading buffer was added to the collected magnetic beads, and the mixture was incubated at 95 °C for 5 min to release the bound proteins. Western Blot analysis was performed using α-GFP (Invitrogen, MA5-15256) and α-HA (Roche, 11867423001) antibodies.

***In vivo/vitro* phosphorylation assay and LC-MS/MS analysis**

The binary vectors pFGC1008-CPK27-HA and pCAMBIA2300-35S:SLAC1-GFP were constructed for the experiment. The primer sequences used for vector construction are listed in Supplemental Table 1. *A. tumefaciens* strains containing the respective vectors were mixed at a 1:1 ratio and used to infect *N. benthamiana* leaves for co-expression for 48 h. Empty vectors were used as negative controls. Proteins were separated by SDS-PAGE and analyzed by Western blot using an anti-pSer/pThr antibody (ECM Biosciences, PM3801).

*In vitro* phosphorylation site identification and LC-MS/MS analysis were conducted following the method described (Hu et al., 2021c). MBP-SLAC1 and His-CPK27 proteins were expressed in *Escherichia coli* for the experiment. The phosphorylation reaction was carried out at room temperature for 3 h, with MBP-SLAC1 as the substrate and His-CPK27 as the kinase. The phosphorylated MBP-SLAC1 was separated by SDS-PAGE and digested with trypsin overnight. Phosphopeptides were analyzed using an LTQ Orbitrap Elite (Thermo Fisher Scientific).

**Assessment of phosphorylation site activity**

Function of SLAC1 phosphorylation sites was assessed using the tobacco transient overexpression system, following the method described by Hu et al. (Hu et al., 2021c). Site-directed mutagenesis vectors were constructed to introduce mutations at serine (Ser, S) and aspartic acid (Asp, D) residues to simulate dephosphorylated and constitutively phosphorylated states, respectively. The primer sequences used for mutagenesis are provided in Supplementary Supplemental Table 1. *A. tumefaciens* strains containing the respective vectors were used to infect *N. benthamiana* leaves, and the plants were incubated for 48 hours. Subsequently, W+OS treatment was applied, and stomatal phenotypes were evaluated at 0, 5, 10, and 15 minutes post-treatment.

**RNA Extraction and** **qRT-PCR Analysis**

The experiment was conducted to verify the silencing efficiency of VIGS. Total RNA was extracted using the RNA prep pure Plant Kit (TIANGEN, Beijing, China) following the instructions provided in the kit. Total RNA (0.5 μg) was reverse transcribed into cDNA using the HiScript II Q RT SuperMix for qPCR Kit (Vazyme Biotech Co., Ltd). The qRT-PCR experiment was performed on the Light Cycler 480 II Real-Time PCR (Roche, Swiss) detection system. The gene-specific primers used in the qRT-PCR analysis are listed in Supplemental Table 2.

**Statistical analysis**

The experiment adopted a completely randomized design with at least three replicates. Data were statistically analyzed using IBM SPSS Statistics 26 software (IBM, USA). One-way ANOVA was used for variance analysis, and the significance of differences among treatments was tested by Tukey's method (*P* < 0.05).

**Accession numbers**

Sequence data of genes mentioned in this study can be found in the Sol Genomics Network (http://solgenomics.net/) database under the following accession numbers: *SLAC1*, Solyc08g079770; *CPK1*, *Solyc01g006730*; *CPK2*, *Solyc01g006840*; *CPK3*, *Solyc01g112250*; *CPK4*, *Solyc04g009800*; *CPK5*, *Solyc04g049160*; *CPK6*, *Solyc05g056570*; *CPK7*, *Solyc06g065380*; *CPK8*, *Solyc10g074570*; *CPK9*, *Solyc10g076900*; *CPK10*, *Solyc10g081640*; *CPK11*, *Solyc10g081740*; *CPK12*, *Solyc11g006370*; *CPK13*, *Solyc11g018610*; *CPK14*, *Solyc01g008740*; *CPK15*, *Solyc02g032820*; *CPK16*, *Solyc03g031670*; *CPK17*, *Solyc04g081910*; *CPK18*, *Solyc07g064610*; *CPK19*, *Solyc08g008170*; *CPK20*, *Solyc11g064900*; *CPK21*, *Solyc12g099790*; *CPK22*, *Solyc01g008440*; *CPK23*, *Solyc03g113390*; *CPK24*, *Solyc06g073350*; *CPK25*, *Solyc09g005550*; *CPK26*, *Solyc10g079130*; *CPK27*, *Solyc11g065660*; *CPK28*, *Solyc02g083850*; *CPK29*, *Solyc03g033540*; *GLR3.3*, *Solyc04g082610*; *GLR3.5*, *Solyc02g082480*; *RBOH1*, *Solyc08g081690*; *MYC2*, *Solyc08g076930*; *ACTIN2*, *Solyc11g005330*; *UBI3*, *Solyc01g056940*.

# Supplemental References

**Hu, C., Wei, C., Ma, Q., Dong, H., Shi, K., Zhou, Y., Foyer, C.H., and Yu, J.** (2021a). Ethylene response factors 15 and 16 trigger jasmonate biosynthesis in tomato during herbivore resistance. Plant Physiology **185**:1182-1197. 10.1093/plphys/kiaa089.

**Hu, C., Wu, S., Li, J., Dong, H., Zhu, C., Sun, T., Hu, Z., Foyer, C.H., and Yu, J.** (2022). Herbivore-induced Ca^2+^ signals trigger a jasmonate burst by activating ERF16-mediated expression in tomato. New Phytologist **236**:1796-1808. 10.1111/nph.18455.

**Hu, C.Y., Duan, S.Q., Zhou, J., and Yu, J.Q.** (2021b). CHARACTERISTICS OF HERBIVORY/WOUND-ELICITED ELECTRICAL SIGNAL TRANSDUCTION IN TOMATO. Frontiers of Agricultural Science and Engineering **8**:292-301. 10.15302/j-fase-2021395.

**Hu, Z., Li, J., Ding, S., Cheng, F., Li, X., Jiang, Y., Yu, J., Foyer, C.H., and Shi, K.** (2021c). The protein kinase CPK28 phosphorylates ascorbate peroxidase and enhances thermotolerance in tomato. Plant Physiology **186**:1302-1317. 10.1093/plphys/kiab120.

**Shi, K., Li, X., Zhang, H., Zhang, G., Liu, Y., Zhou, Y., Xia, X., Chen, Z., and Yu, J.** (2015). Guard cell hydrogen peroxide and nitric oxide mediate elevated CO_2_-induced stomatal movement in tomato. New Phytologist **208**:342-353. 10.1111/nph.13621.

**Yang, X., Baliji, S., Buchmann, R.C., Wang, H., Lindbo, J.A., Sunter, G., and Bisaro, D.M.** (2007). Functional modulation of the geminivirus AL2 transcription factor and silencing suppressor by self-interaction. Journal of Virology **81**:11972-11981. 10.1128/jvi.00617-07.

**Zheng, X.L., Yang, H.F., Zou, J.P., Jin, W.D., Qi, Z.Y., Yang, P., Yu, J.Q., and Zhou, J.** (2024). SnRK1α1-mediated RBOH1 phosphorylation regulates reactive oxygen species to enhance tolerance to low nitrogen in tomato. Plant Cell **37**10.1093/plcell/koae321.

**Zhu, C., Hu, Z., Hu, C., Ma, H., Zhou, J., Xia, X., Shi, K., Foyer, C.H., Yu, J., and Zhou, Y.** (2024). SlCPK27 cross-links SlHY5 and SlPIF4 in brassinosteroid-dependent photo- and thermo-morphogenesis in tomato. Proceedings of the National Academy of Sciences of the United States of America **121**:e2403040121. 10.1073/pnas.2403040121.

# Supplemental Figures


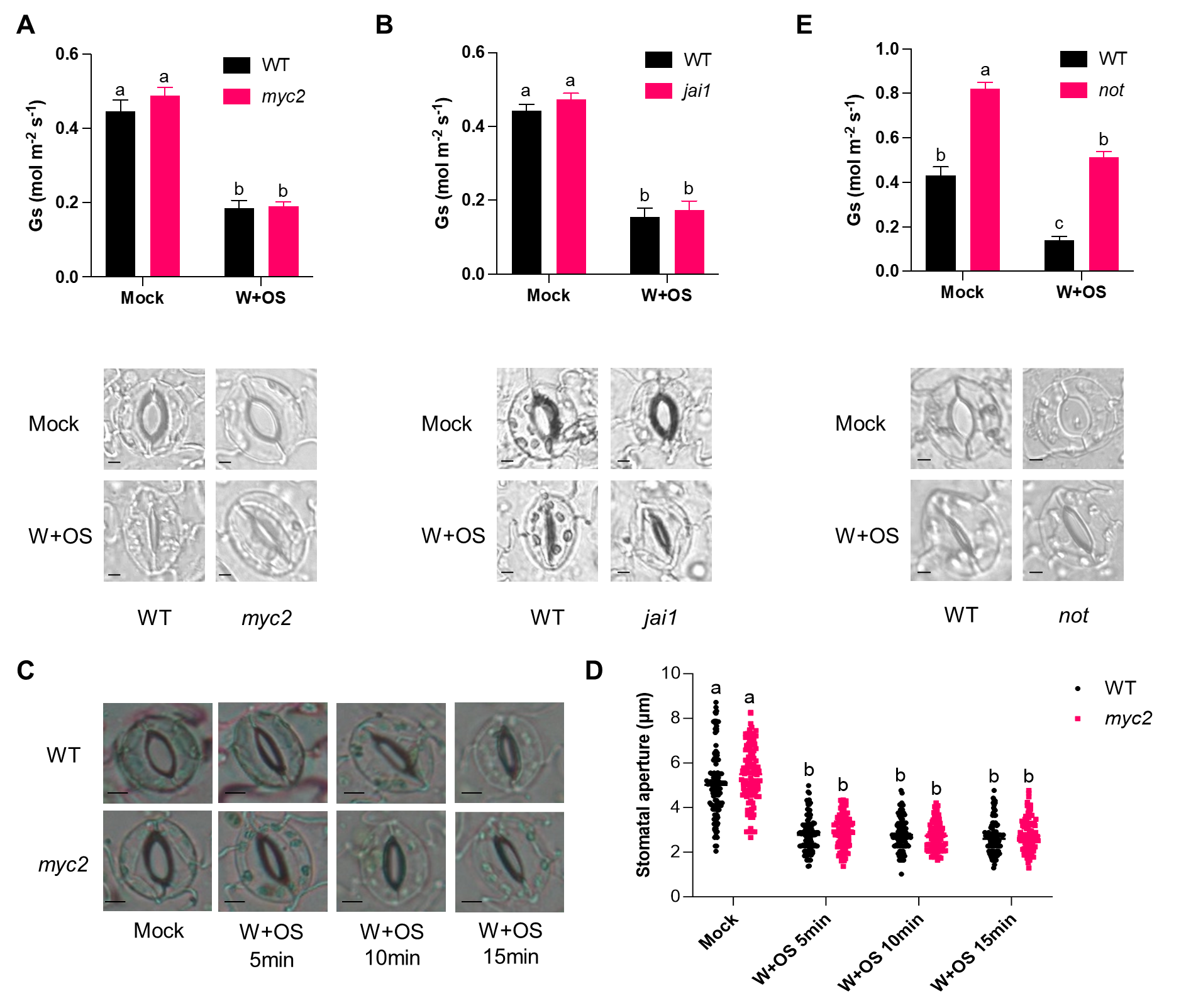


**Supplemental Fig. 1 Effects of JA and ABA on the stomatal aperture of tomato leaves by herbivory.**

(A-B) Stomatal conductance and representative images of WT vs. *myc2* (A) and *jai1* (B) after W+OS for 15min. Aperture measurements shown in (Fig1 G-H). Data are mean ± SD (n = 6 for conductance; n > 100 for aperture). Scale bars = 5 μm. (C) Representative stomatal images of WT and *myc2* after W+OS for 5, 10, 15 min. Scale bar = 5 μm. (D) Stomatal aperture of WT and *myc2* after W+OS for 5, 10, 15 min. Data represent the mean ± SD (n > 100). (E) Stomatal conductance and representative images of WT and *not* after W+OS. Aperture measurements shown in (Fig1 I). Data are mean ± SD (n = 6 for conductance; n > 100 for aperture). Scale bars = 5 μm. Different letters indicate significant differences (*P* < 0.05, Tukey’s test).


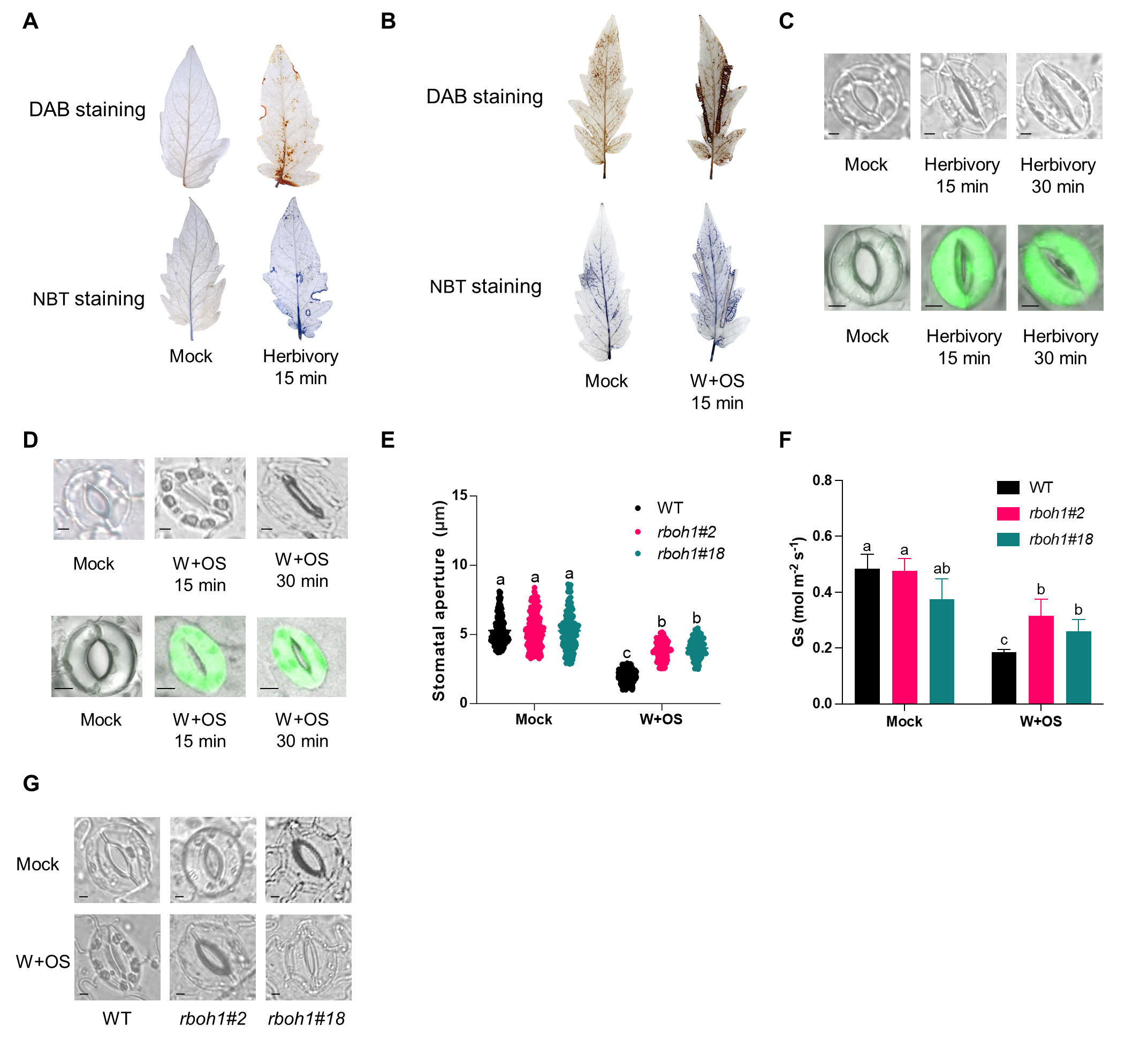


**Supplemental Fig. 2 Effects of cotton bollworm feeding on the stomatal aperture and ROS accumulation in tomato.**

(A-B) *H. armigera* feeding and W+OS-induced ROS accumulation in leaf tissue. (C) Representative stomatal images after herbivory (top) and *H*. *armigera* feeding-induced ROS accumulation in guard cells of the lower epidermis of the leaf (bottom). Scale bar = 5 μm. (D) Representative images of stomatal apertures (top) and ROS accumulation in guard cells (bottom) at indicated time points following W+OS treatment. Scale bar = 5 μm. (E) Stomatal aperture of WT and *rboh1* tomato leaves after W+OS. Data represent the mean ± SD (n > 100). (F) Stomatal conductance of WT and *rboh1* tomato leaves after W+OS. Data represent the mean ± SD (n = 6). (G) Representative stomatal images of WT and *rboh1* after W+OS. Scale bar = 5 μm. Different letters indicate significant differences between treatments (*P* < 0.05, Tukey's test).


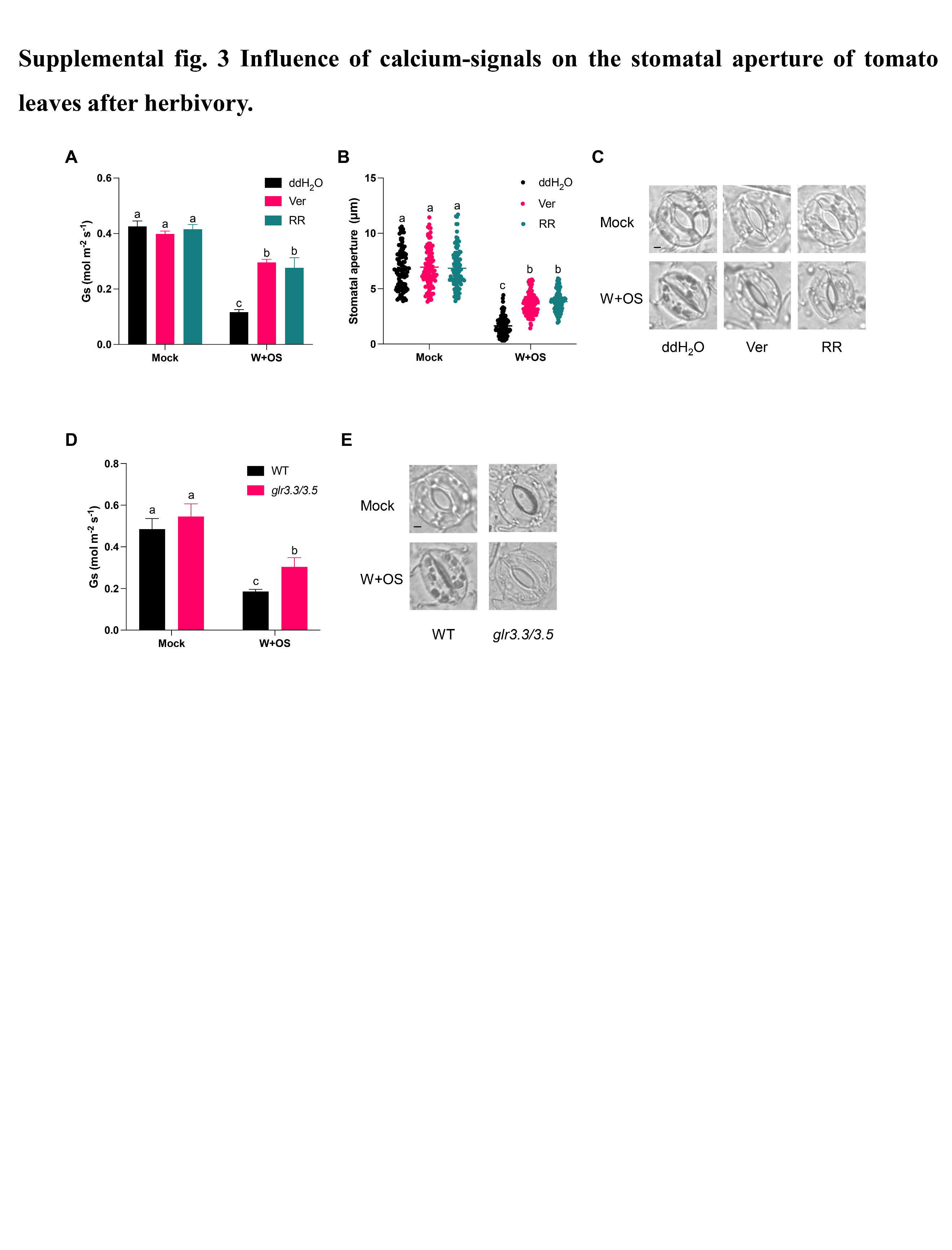


**Supplemental Fig. 3 Influence of calcium-signals on the stomatal aperture of tomato leaves after** **herbivory.**

(A) Effects of ruthenium red (RR) and verapamil (Ver) treatments on stomatal conductance of WT tomato leaves in response to W+OS. Data represent the mean ± SD (n = 6). (B) Effects of RR and Ver treatments on stomatal aperture of WT tomato leaves in response to W+OS. Data represent the mean ± SD (n > 100); (C) Representative stomatal images of WT under RR and Ver treatments after W+OS. Scale bar = 5 μm. Different letters indicate significant differences between treatments (*P* < 0.05, Tukey's test). (D) Stomatal conductance of WT and *glr3.3/3.5* tomato leaves after W+OS. Data represent the mean ± SD (n = 6). (E) Representative stomatal images of WT and *glr3.3/3.5* after W+OS. Scale bar = 5 μm. Different letters indicate significant differences between treatments (*P* < 0.05, Tukey's test).


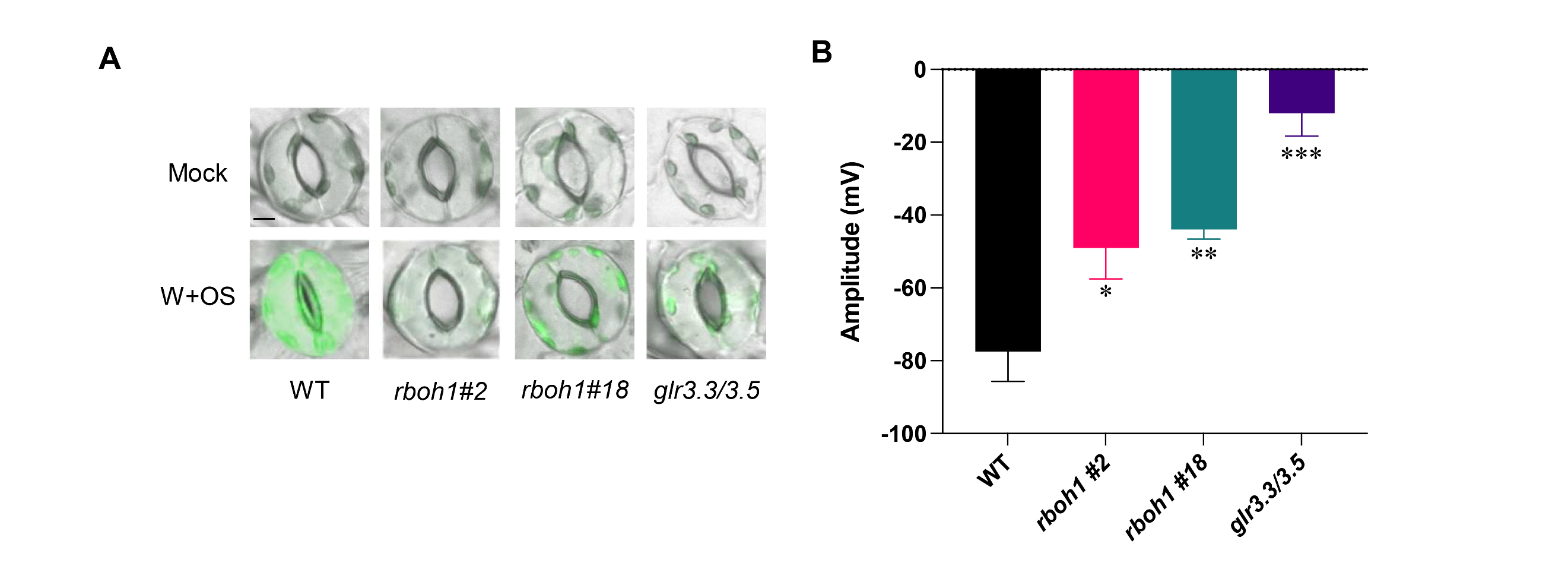


**Supplemental Fig. 4 Impact of herbivory on the accumulation of ROS and electrical signals in WT, *rboh1* and *glr3.3/3.5* tomato.**

(A) Accumulation of ROS in guard cells at 15 min after W+OS treatment. Scale bar = 5 μm. (B) The amplitude of the wounding-induced electrical signals. Data represent the mean ± SD (n = 6). Statistically significant differences are indicated using asterisks (**P* < 0.05, ***P* < 0.01, ****P* <0.001, one-way ANOVA followed by Tukey test).


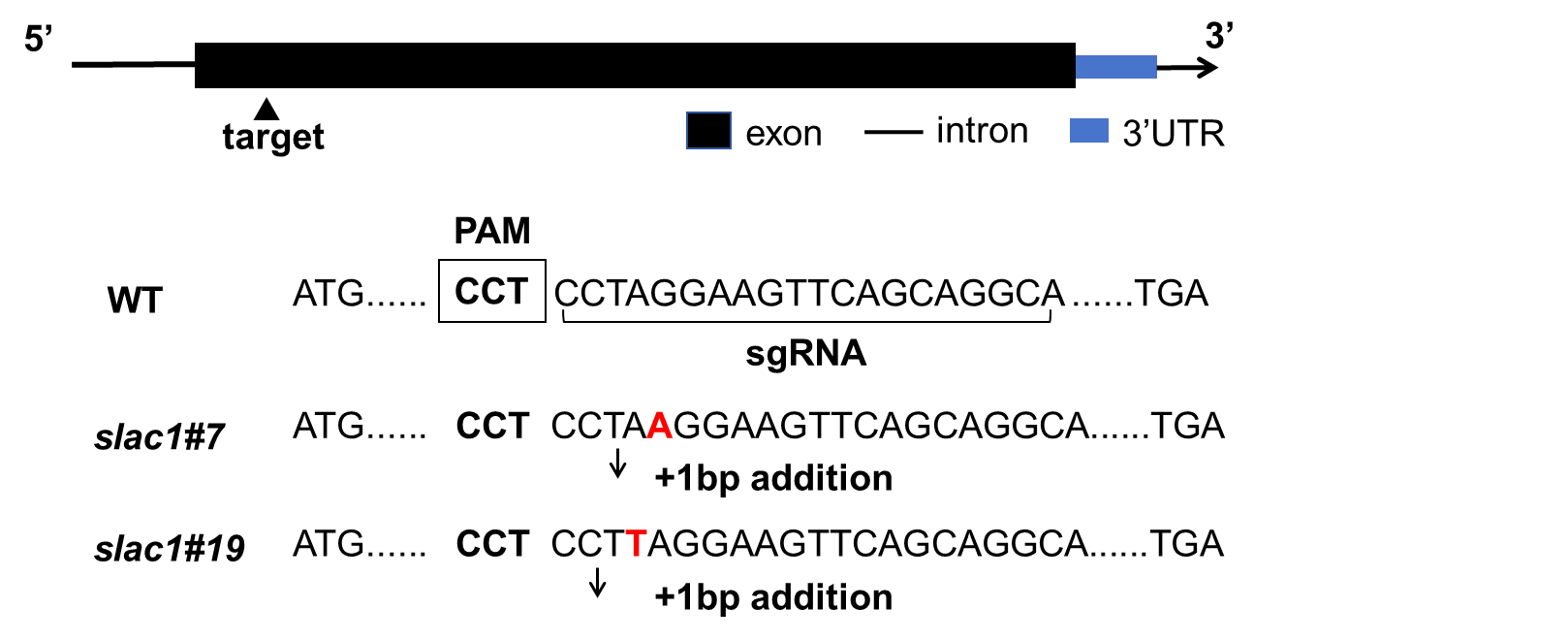


**Supplemental Fig. 5 The role of SLAC1 in the stomatal movement of tomato leaves induced by herbivory.**

Information on tomato plants of the *slac1* knockout lines and sanger sequencing analysis. PAM is indicated by the box.


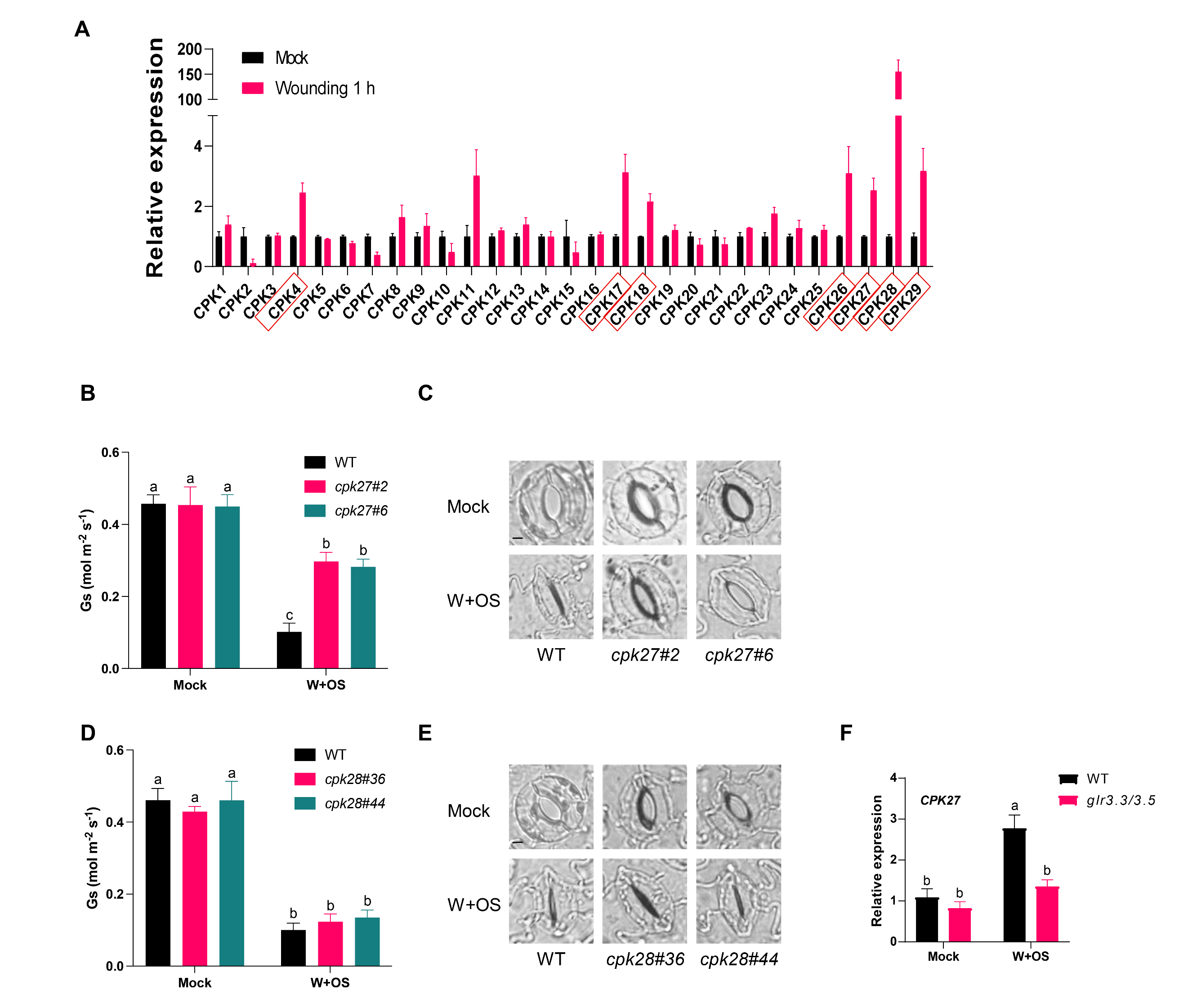


**Supplemental Fig. 6 CPK27 is involved in the influence herbivory on the stomatal aperture.**

1. RT-qPCR analysis of the expression of *CPK* family in tomato 1 h after wounding treatment. *ACTIN2* and *UBI3* were used as reference genes. Relative expression was calculated against the expression level in Mock, which was designated as 1. Data are shown as mean ± SD (n = 3). (B) Stomatal conductance of WT and *cpk27* tomato leaves after W+OS. Data represent the mean ± SD (n = 6). (C) Representative stomatal images of WT and *cpk27* after simulated herbivory. Scale bar = 5 μm. (D) Stomatal conductance of WT and *cpk28* tomato leaves after W+OS. Data represent the mean ± SD (n = 6). (E) Representative stomatal images of WT and *cpk28* after simulated herbivory. Scale bar = 5 μm. (F) RT-qPCR analysis of *CPK27* expression in *glr3.3/3.5* mutants and WT plants after W+OS. *ACTIN2* and *UBI3* were used as reference gene. Relative expression was calculated against the expression level in Mock (WT), which was designated as 1. Data are shown as mean ± SD (n = 3). Different letters indicate significant differences between treatments (*P* < 0.05, Tukey's test).


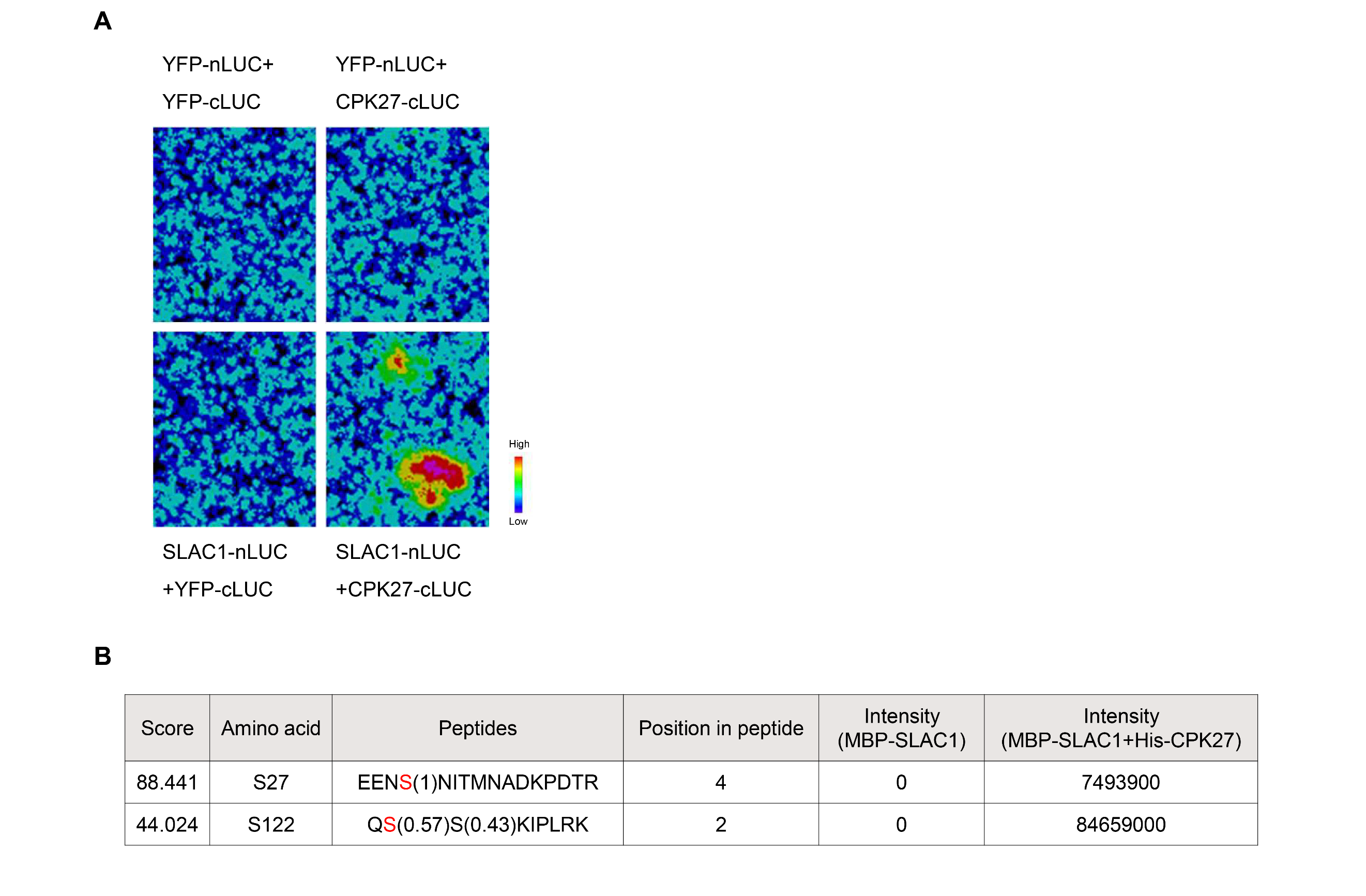


**Supplemental Fig. 7 CPK27 interacts with SLAC1 and phosphorylates SLAC1.**

(A) Split-luciferase imaging assays showing that CPK27 interacts with SLAC1. (B) Summary table of the two SLAC1 phosphosites identified by in vitro phosphorylation reaction followed by LC-MS/MS analysis.

# Supplemental Table 1

**Supplemental Table 1 Primer sequences used for vector construction**

| **ID** | **Primer sequences (**5’ - 3’**)** |
| --- | --- |
| nLUC-SLAC1-F | GAGCTCGGTACCCGGGATCCATGAATGTAGGAAACAACCATTTAG |
| nLUC-SLAC1-R | GGGACGCGTACGAGATCTGGTCGACTTTCTCCTCCTCAGAGGCGCACTCT |
| p2YN-SLAC1-F | CCATTTACGAACGATAGTTAATTAAATGAATGTAGGAAACAACCATTTAG |
| p2YN-SLAC1-R | ACCACTGCCACCTCCTCCACTAGTTTTCTCCTCCTCAGAGGCGCA |
| GFP-SLAC1-F | ATCTCTCTCGAGCTTTCGCGAGCTCATGAATGTAGGAAACAACCATTTAG |
| GFP-SLAC1-R | CCTCGCCCTTGCTCACCATGGATCCTTTCTCCTCCTCAGAGGCGCACTCT |
| ToA-SLAC1-F | GATCACAAGGAAGAAAACGCTAACATTACGATGAATGCTGATAAACCAGACAC GCG |
| ToA-SLAC1-R | CGCGTGTCTGGTTTATCAGCATTCATCGTAATGTTAGCGTTTTCTTCCTTGTGATC |
| ToD-SLAC1-F | GATCACAAGGAAGAAAACGACAACATTACGATGAATGCTGATAAACCAGACAC GCG |
| ToD-SLAC1-R | CGCGTGTCTGGTTTATCAGCATTCATCGTAATGTTGTCGTTTTCTTCCTTGTGATC |
| MBP-SLAC1-F | GGGATCGAGGGAAGGATTTCAGAATTCATGAATGTAGGAAACAACCATTTAG |
| MBP-SLAC1-R | CCTGCAGGTCGACTCTAGAGGATCCTCAAAAAGGCCATTTTTCATCTTTT |
| cLUC-CPK27-F | cgcgtcccggggcGGTACCCATGGGGAATTGCTGTGGGACACCTG |
| cLUC-CPK27-R | CGGGCCCTCTAGAGGATCCCTCATCTACCTTCTTCCTTTCCGACT |
| p2YC-CPK27-F | CCATTTACGAACGATAGTTAATTAACATGGGGAATTGCTGTGGGACACCTG |
| p2YC-CPK27-R | CACCACTGCCACCTCCTCCACTAGTTCTACCTTCTTCCTTTCCGACTTGT |
| His-CPK27-F | CAAGGCCATGGCTGATATCGGATCCATGGGGAATTGCTGTGGGACACCTGG |
| His-CPK27-R | GCAAGCTTGTCGACGGAGCTCGAATTCTCTACCTTCTTCCTTTCCGACTTG |
| cLUC-CPK4-F | ACGCGTCCCGGGGCGGTACCATGGGGAACACTTGTGTTGGACCTA |
| cLUC-CPK4-R | CCGGGCCCTCTAGAGGATCCCTAAAGTTTTAGAACGTCTCTAAAT |
| cLUC-CPK17-F | cgcgtcccggggcGGTACCCTCCTTTTTCTCAAAAAAAATAAAAA |
| cLUC-CPK17-R | CGGGCCCTCTAGAGGATCCCTCATCTTGGCTTCACTTCATCAACA |
| cLUC-CPK18-F | cgcgtcccggggcGGTACCCATGGGTATTTGTGCTAGTAAAGGTA |
| cLUC-CPK18-R | CGGGCCCTCTAGAGGATCCCCTAGAAGACCTTGCCTGGTTGTTTG |
| cLUC-CPK26-F | cgcgtcccggggcGGTACCCATGGGTAATTGTTGTGCAGTACCAA |
| cLUC-CPK26-R | CGGGCCCTCTAGAGGATCCCTTAGCTTTGTAAGGATCCATCCTTC |
| cLUC-CPK28-F | cgcgtcccggggcGGTACCCATGGGCAACATATGTTTTTCTAGCT |
| cLUC-CPK28-R | CGGGCCCTCTAGAGGATCCCCTAAATTTTGCGAGAGCCTCTGACC |
| cLUC-CPK29-F | cgcgtcccggggcGGTACCCATGGGTAGTTGTTTTTCAAGCTCCA |
| cLUC-CPK29-R | CGGGCCCTCTAGAGGATCCCTTACATTCCCCGTGAATCTCTCACC |

# Supplemental Table 2

**Supplemental Table 2 Primer sequences used for qRT-PCR analysis**

| **ID** | **Primer sequences (**5’-3’**)** |
| --- | --- |
| qRT-PCR-ACTIN2-F | GGTGTGATGGTGGGTATGG |
| qRT-PCR-ACTIN2-R | GCTGACAATTCCGTGCTC |
| qRT-PCR-UBI3-F | AGTCCACTCTCCATCTCGTG |
| qRT-PCR-UBI3-R | CTCAGCATTAGGGCACTCCT |
| qRT-PCR-CPK1-F | GACAAGGTCAATTCGGGACT |
| qRT-PCR-CPK1-R | CTGCCAAATGGTGCATAATC |
| qRT-PCR-CPK2-F | TGCAGTTCATGTTGTGATGG |
| qRT-PCR-CPK2-R | AAGCTCAGCAGCCTGTCTCT |
| qRT-PCR-CPK3-F | CCGAATTGTCTTCCGGTATC |
| qRT-PCR-CPK3-R | ACCCTTGATGGAAACGATGT |
| qRT-PCR-CPK4-F | TCTGTTTCAGCTGCAATGTG |
| qRT-PCR-CPK4-R | TCAGGGTCTTTCCCAGATTC |
| qRT-PCR-CPK5-F | TGCTTGACAGGAATCCAAAG |
| qRT-PCR-CPK5-R | CTTTGGATTCCTGTCAAGCA |
| qRT-PCR-CPK6-F | TCAGATACTGCGAGGCAAAC |
| qRT-PCR-CPK6-R | TGGGCAGTTAACCTCCTCTT |
| qRT-PCR-CPK7-F | AGGATCCTAAAGCGCGAATA |
| qRT-PCR-CPK7-R | ATTCAACACTGCAGATCCCA |
| qRT-PCR-CPK8-F | TGGCCGTTGATATCTGAGAG |
| qRT-PCR-CPK8-R | TACTGCAGGATCCAGTGCTC |
| qRT-PCR-CPK9-F | AAGTCCTGCGAAAGCGTTAT |
| qRT-PCR-CPK9-R | CTTGCTCAGTTTCATCCCAA |
| qRT-PCR-CPK10-F | TTCAACTCTGAAGGCGATTG |
| qRT-PCR-CPK10-R | GGCTTCCAACAACATCATTG |
| qRT-PCR-CPK11-F | GCGGGTATATTACCGCAGAT |
| qRT-PCR-CPK11-R | TCCGTCCATCGTTATCTTGA |
| qRT-PCR-CPK12-F | AGCTGATGTCTGGAGTGCTG |
| qRT-PCR-CPK12-R | AAGTCAAGATCACCGTGCAG |
| qRT-PCR-CPK13-F | CTCACCAGGGATCCTAGAGC |
| qRT-PCR-CPK13-R | GACAGAACTGCAGAACCCAA |
| qRT-PCR-CPK14-F | TGGAGAAGCACCAGACAC |
| qRT-PCR-CPK14-R | AACATCCTGCAATAACCC |
| qRT-PCR-CPK15-F | ACGGACAATAGTGGGACA |
| qRT-PCR-CPK15-R | TGCTTAACTTCAGCCTCC |
| qRT-PCR-CPK16-F | TGCTGGAGGAGAGTTGTTTG |
| qRT-PCR-CPK16-R | TTCTCTGGCTTGAGATCCCT |
| qRT-PCR-CPK17-F | CTTAGACACGCGATGGAAGA |
| qRT-PCR-CPK17-R | CGCGACAAACTCATCGTAGT |
| qRT-PCR-CPK18-F | AGAAGTGGAGCCAAACAACC |
| qRT-PCR-CPK18-R | CAGAGCTCATCCTGCACATT |
| qRT-PCR-CPK19-F | AGAGTCGGAAGTCAGGCAGT |
| qRT-PCR-CPK19-R | TGATCTTCTCTTTCCATGCG |
| qRT-PCR-CPK20-F | CATCTTTACACAGCATTCCAA |
| qRT-PCR-CPK20-R | TATTTGAGTCAACTTCAGCA |
| qRT-PCR-CPK21-F | GGAAACAATGACATGCAAGG |
| qRT-PCR-CPK21-R | TGGCTTTGAAGAATGGTTTG |
| qRT-PCR-CPK22-F | TACAGACAAGGATGGTCGGA |
| qRT-PCR-CPK22-R | ATGAGCCTTCCCTCATCAAC |
| qRT-PCR-CPK23-F | CATGGCCTCAAGTCTCTGAA |
| qRT-PCR-CPK23-R | TCTCCCAGTGGAACATTTGA |
| qRT-PCR-CPK24-F | ATGGGACAAACTGTTGCTGA |
| qRT-PCR-CPK24-R | TTGTAAGTGCACAGCCATTG |
| qRT-PCR-CPK25-F | CTGCTGTTACACGGACGATT |
| qRT-PCR-CPK25-R | TTCTCGGGCTTCAAATCTCT |
| qRT-PCR-CPK26-F | TGATTGCTGAGCATTTGACA |
| qRT-PCR-CPK26-R | GGAATTTGATGGCCAAGTTT |
| qRT-PCR-CPK27-F | GCAGCTGCGGTTATTATGAA |
| qRT-PCR-CPK27-R | GCCTTCAAAGGAGCAGTTTC |
| qRT-PCR-CPK28-F | AGTTGTTGGGTCATGGACAA |
| qRT-PCR-CPK28-R | CTGCAATTGGAACAACCATC |
| qRT-PCR-CPK29-F | TGGGTGGAGAGTTAGGAAGG |
| qRT-PCR-CPK29-R | AGGCGCTTGTCGATAGACTT |
